# Supplementary figures and images for: The effects of mycobacterial RmlA perturbation on cellular dNTP pool, cell morphology, and replication stress in Mycobacterium smegmatis
Source: PLoS One. 2022 Feb 24;17(2):e0263975. doi: 10.1371/journal.pone.0263975 (PMC8870461; doi:10.1371/journal.pone.0263975)

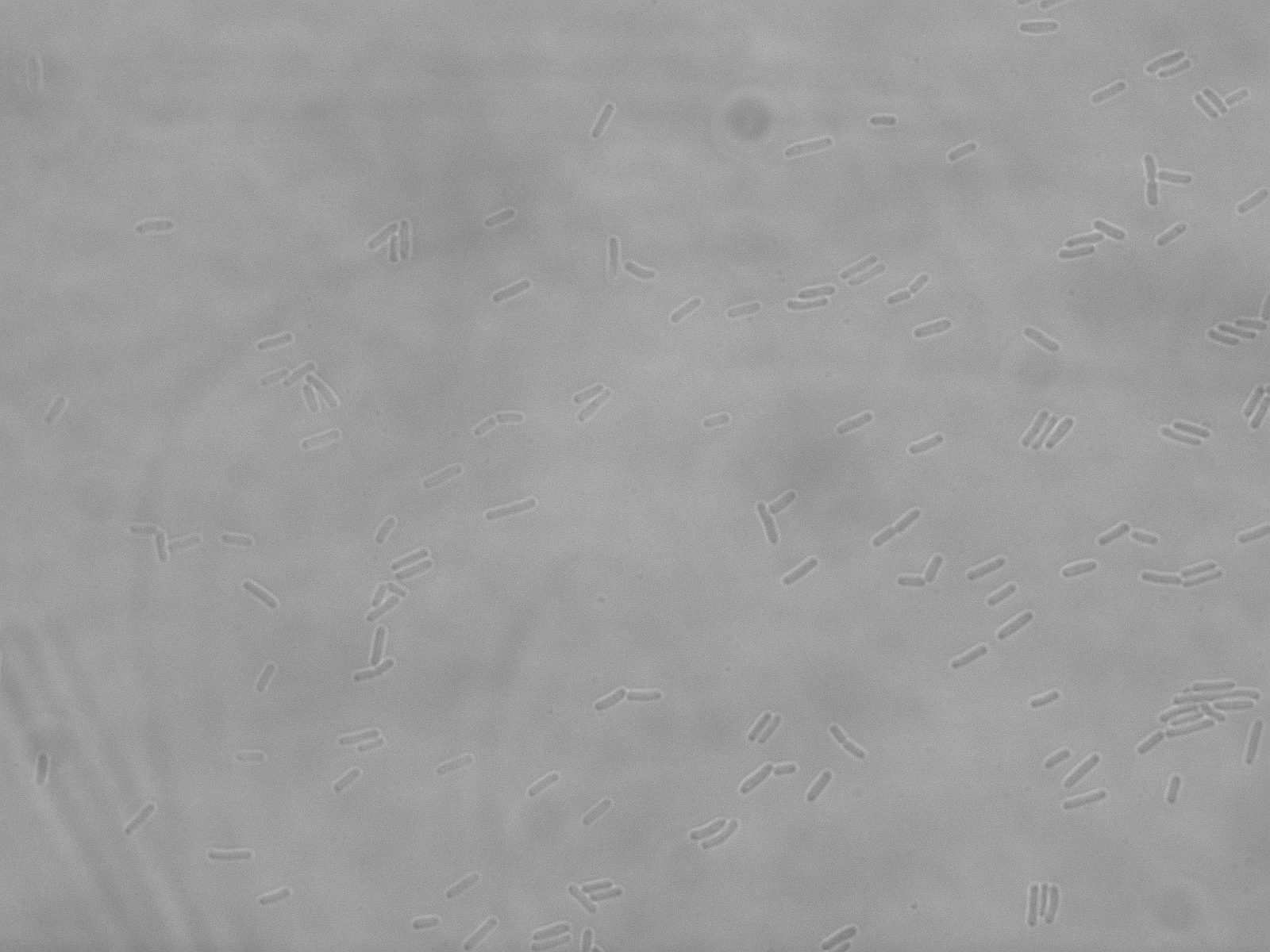

Supplement: S1 File — (ZIP) [file pone.0263975.s001.zip › raw data/microscopy/clumps/dpkng_noind3.tif]

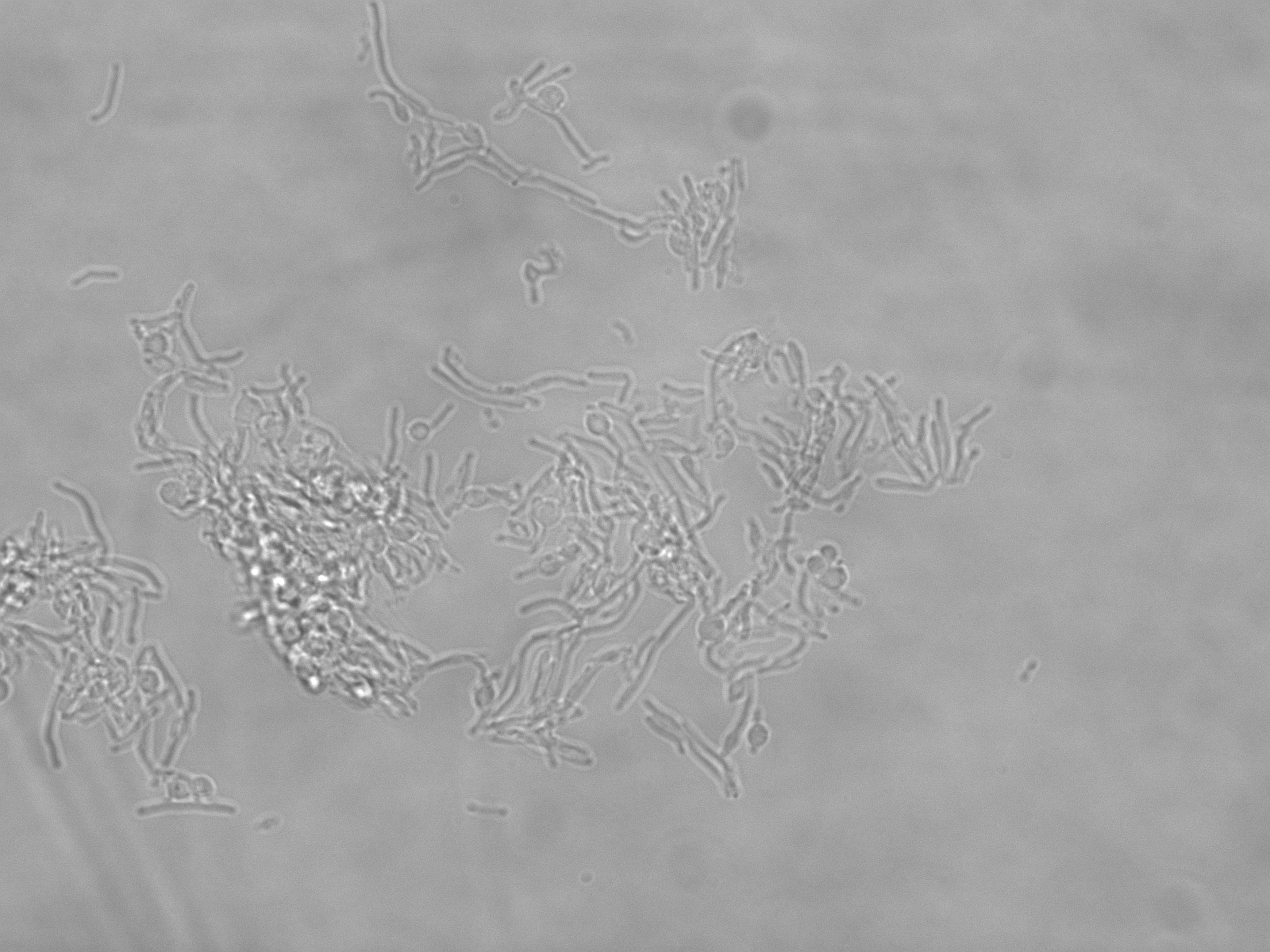

Supplement: S1 File — (ZIP) [file pone.0263975.s001.zip › raw data/microscopy/clumps/dpkng_rmla_tc7.tif]

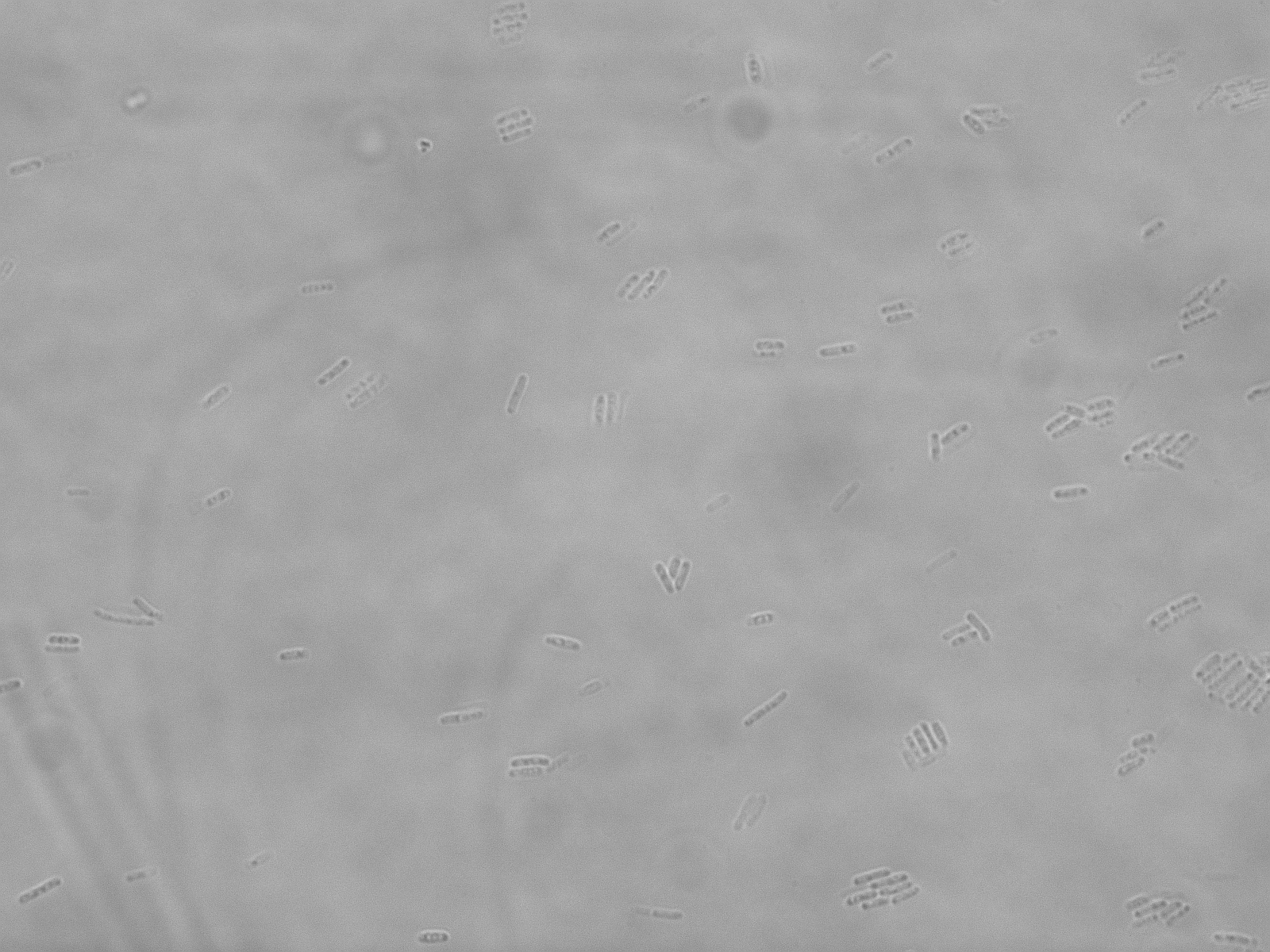

Supplement: S1 File — (ZIP) [file pone.0263975.s001.zip › raw data/microscopy/clumps/dpkng_tc2.tif]

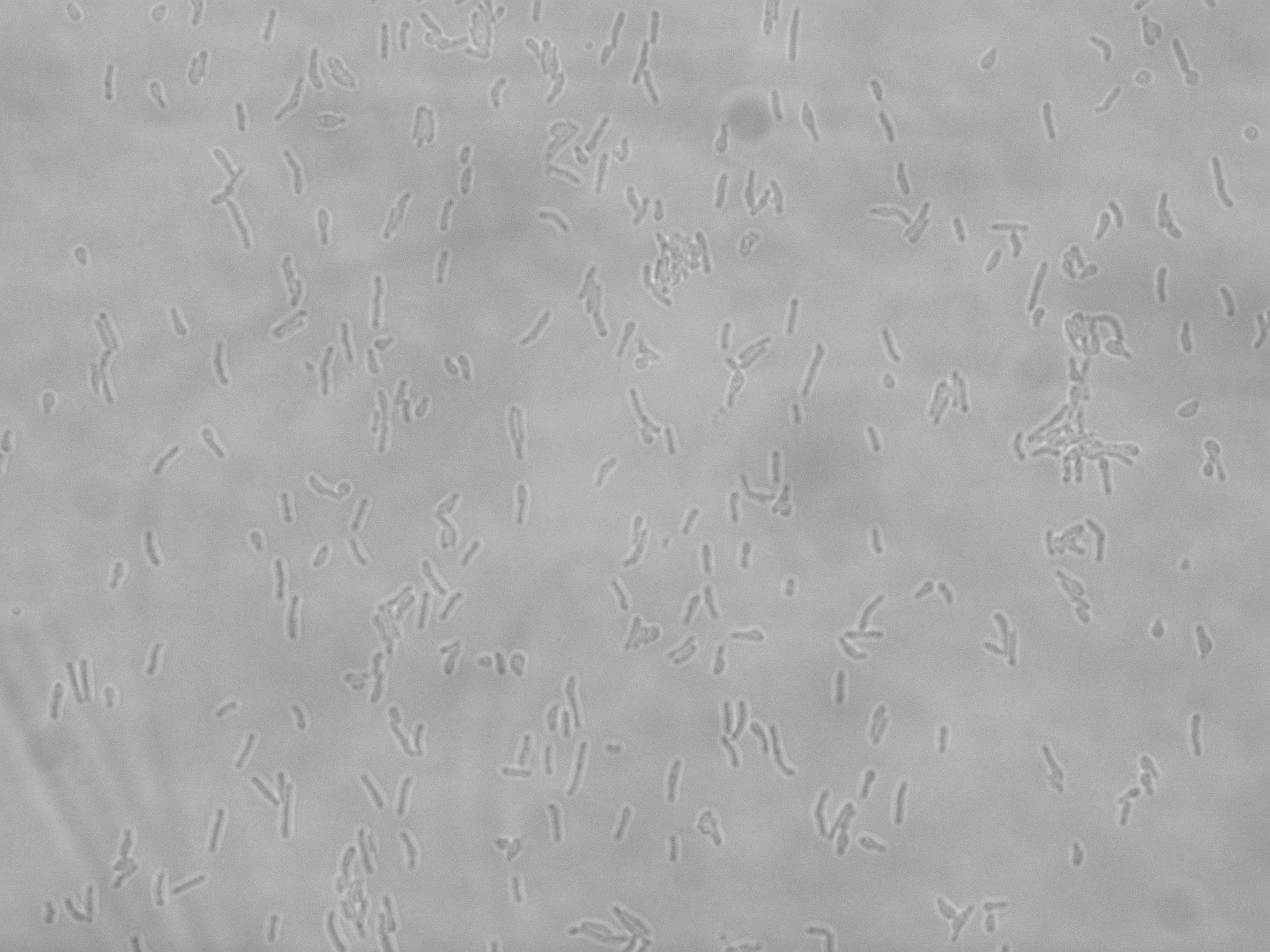

Supplement: S1 File — (ZIP) [file pone.0263975.s001.zip › raw data/microscopy/clumps/RmlA-over_noind_pkng2.tif]

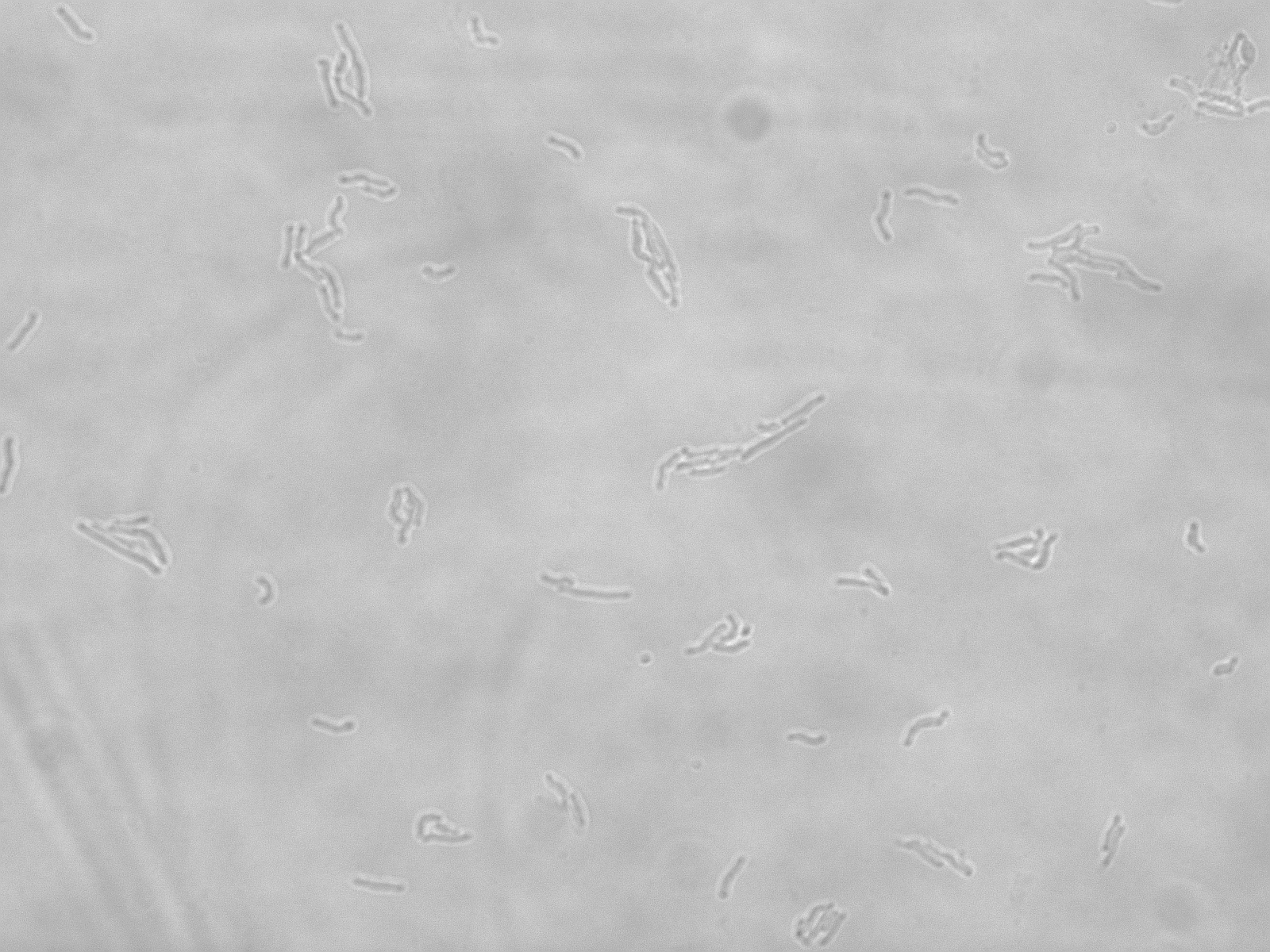

Supplement: S1 File — (ZIP) [file pone.0263975.s001.zip › raw data/microscopy/clumps/rmla_wt_noind2.tif]

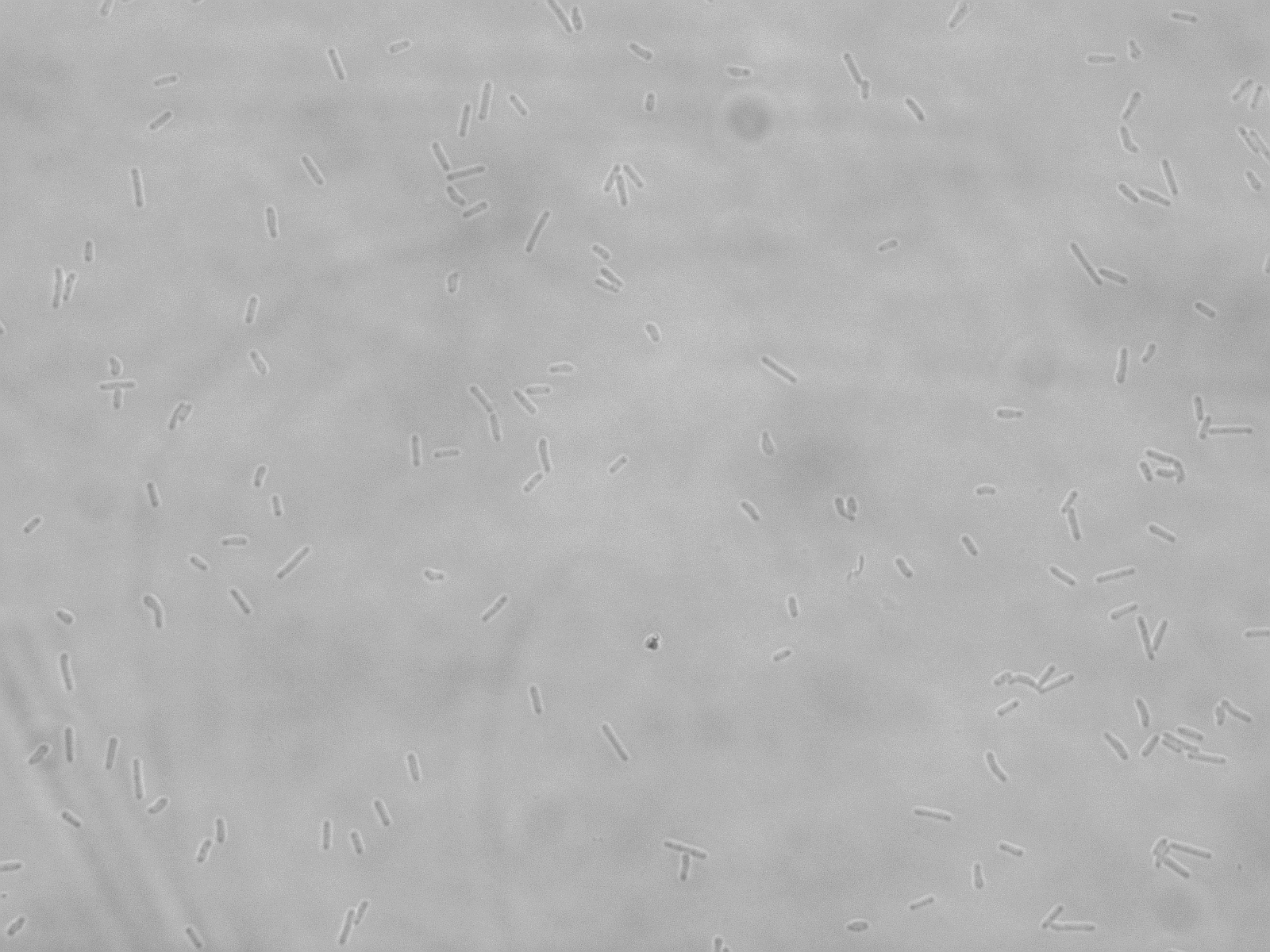

Supplement: S1 File — (ZIP) [file pone.0263975.s001.zip › raw data/microscopy/clumps/wt3.tif]

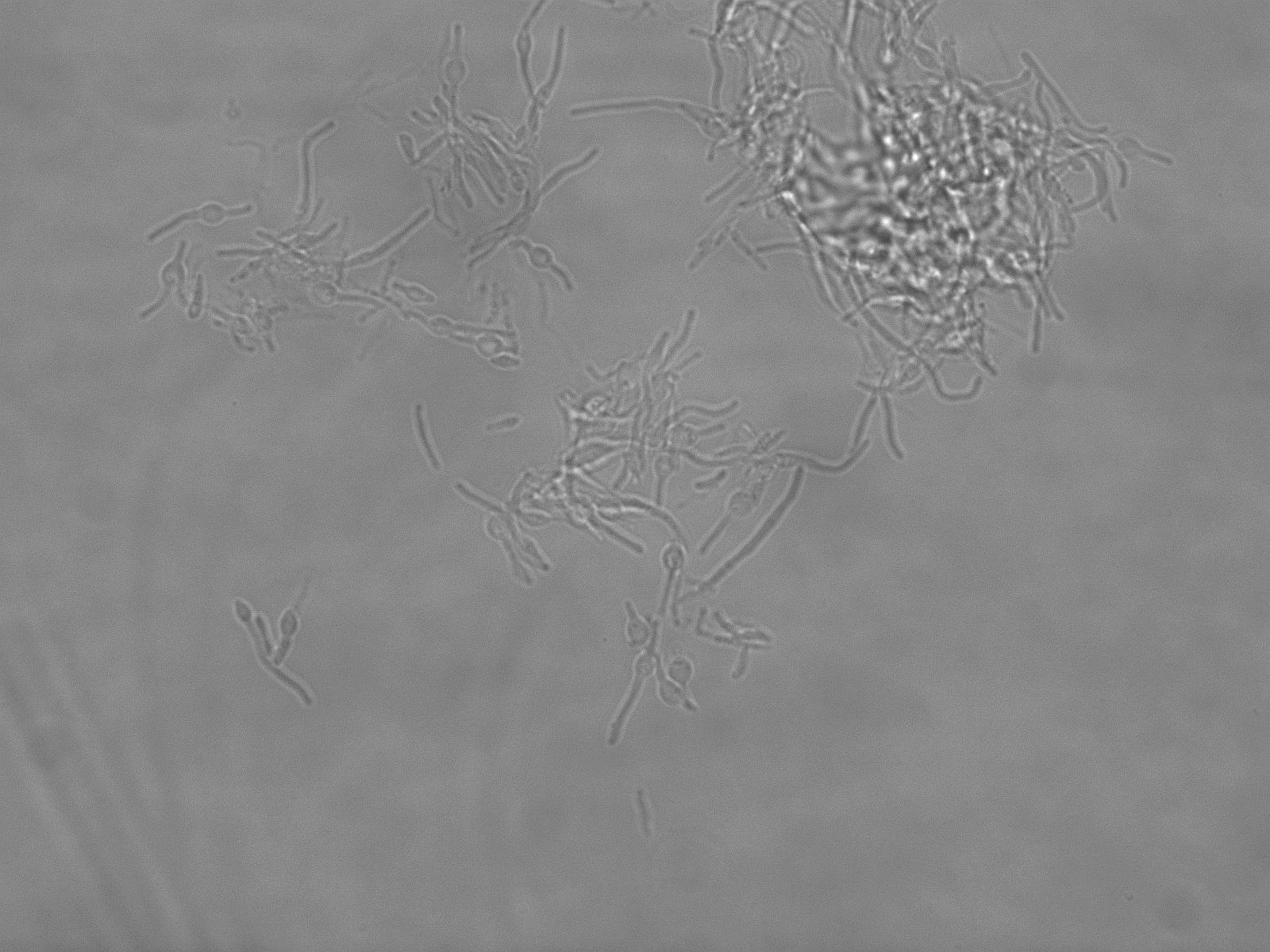

Supplement: S1 File — (ZIP) [file pone.0263975.s001.zip › raw data/microscopy/clumps/wt_rmlA_tc_.tif]

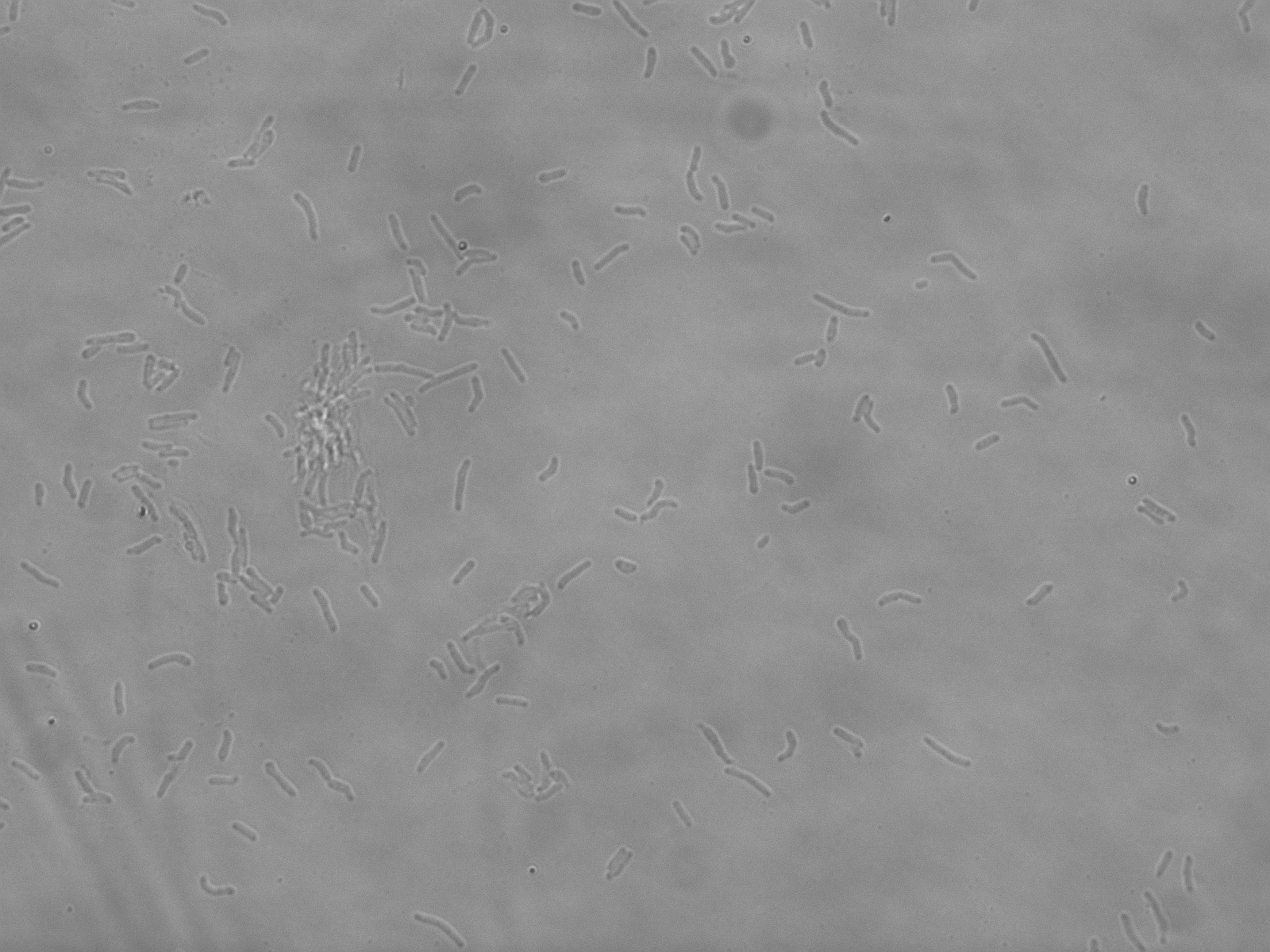

Supplement: S1 File — (ZIP) [file pone.0263975.s001.zip › raw data/microscopy/clumps/wt_tc2.tif]

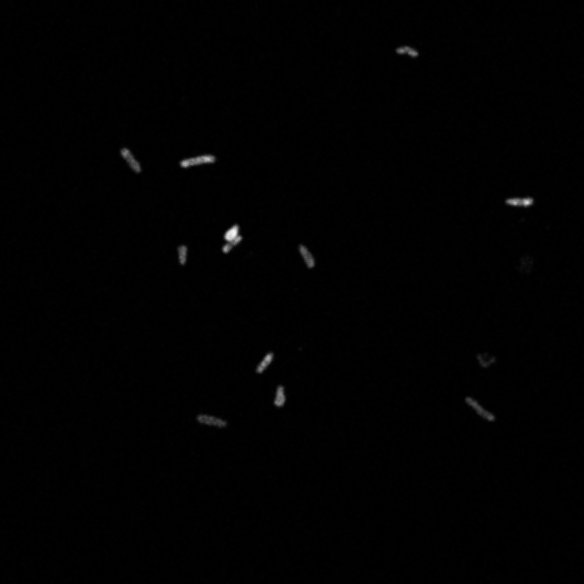

Supplement: S1 File — (ZIP) [file pone.0263975.s001.zip › raw data/microscopy/localization/mOrange2_control.jpg]

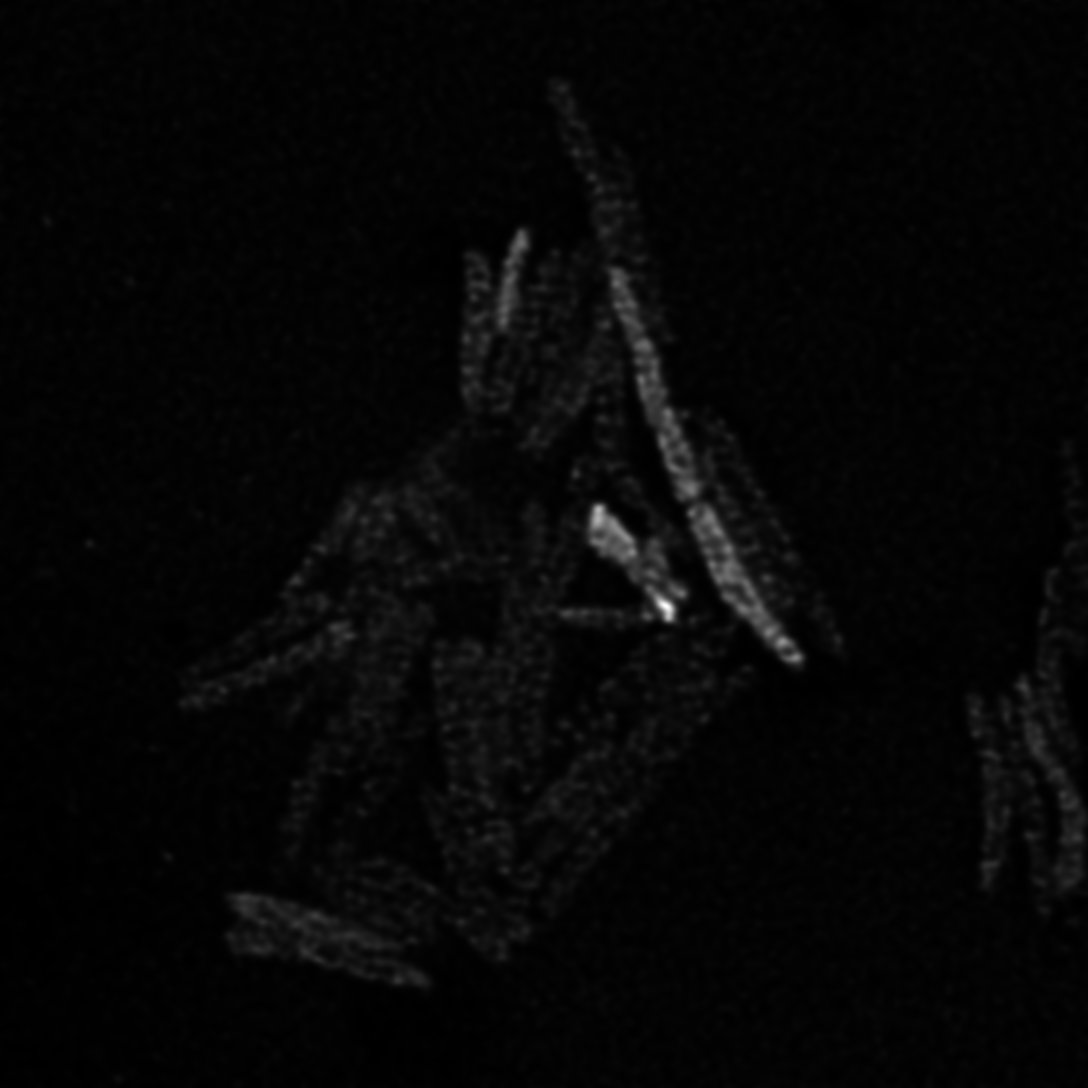

Supplement: S1 File — (ZIP) [file pone.0263975.s001.zip › raw data/microscopy/localization/Rmla_7_decon.jpg]

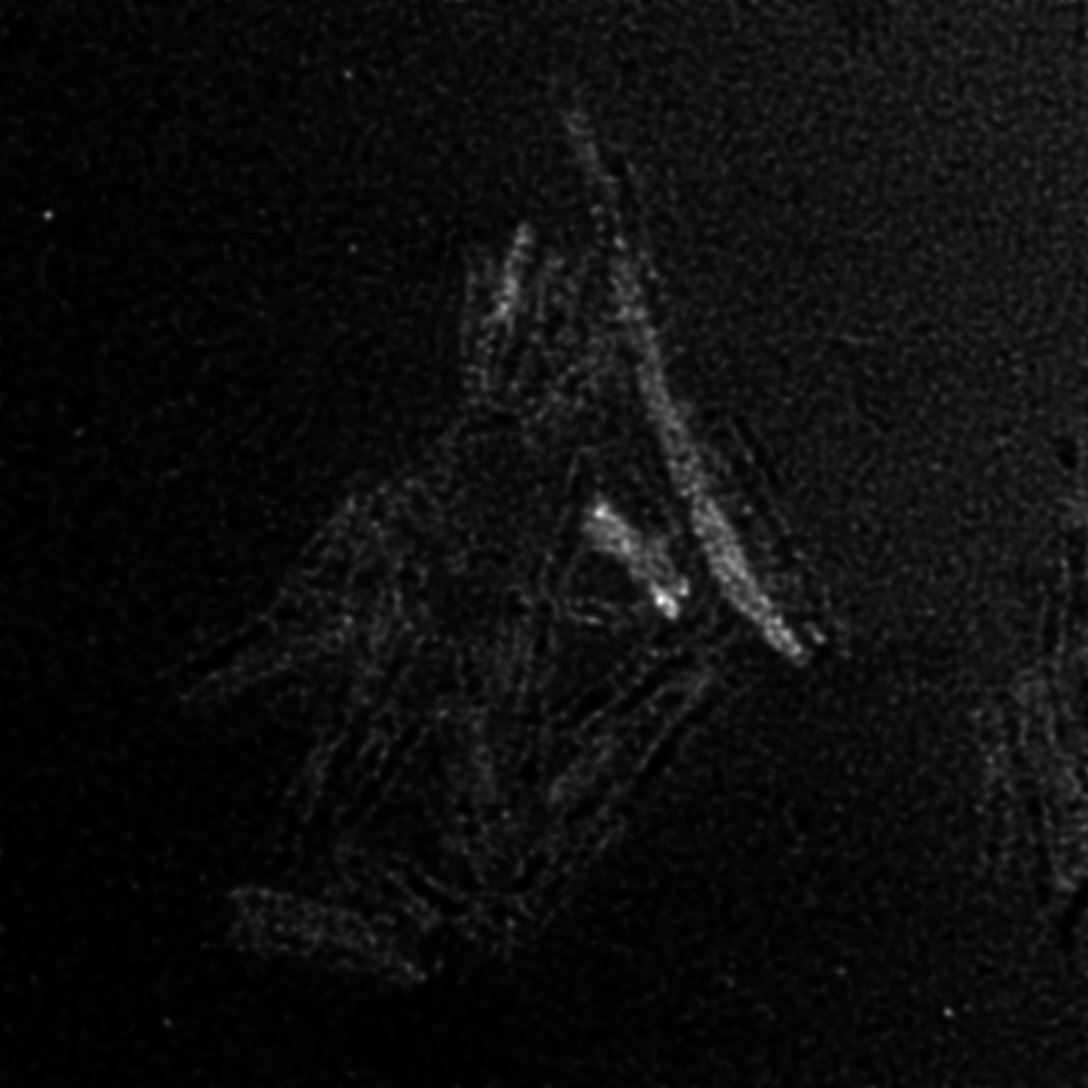

Supplement: S1 File — (ZIP) [file pone.0263975.s001.zip › raw data/microscopy/localization/Rmla_8_decon.jpg]

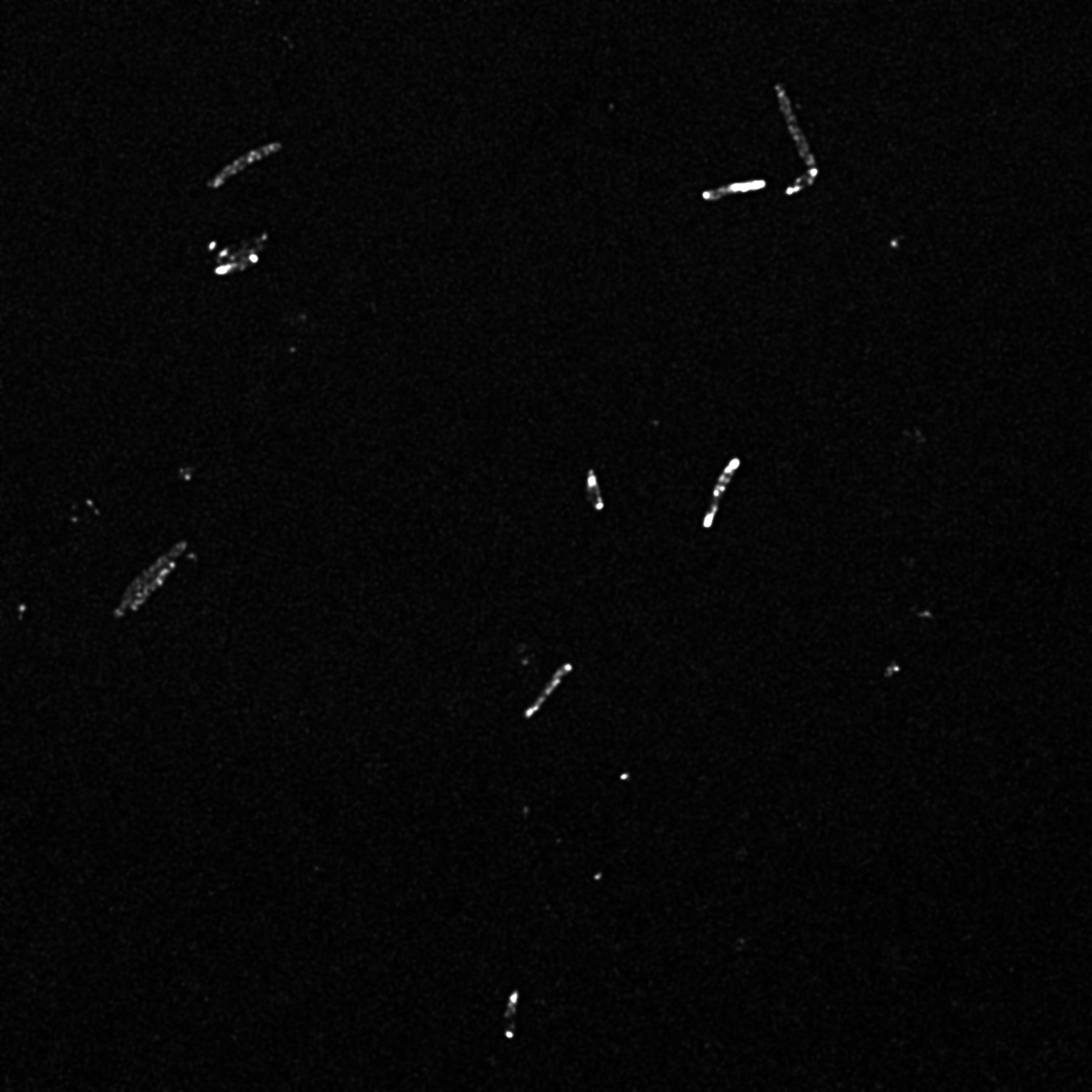

Supplement: S1 File — (ZIP) [file pone.0263975.s001.zip › raw data/microscopy/localization/RmlA_GFP_z_2_decon - Z=10 C=0.jpg]

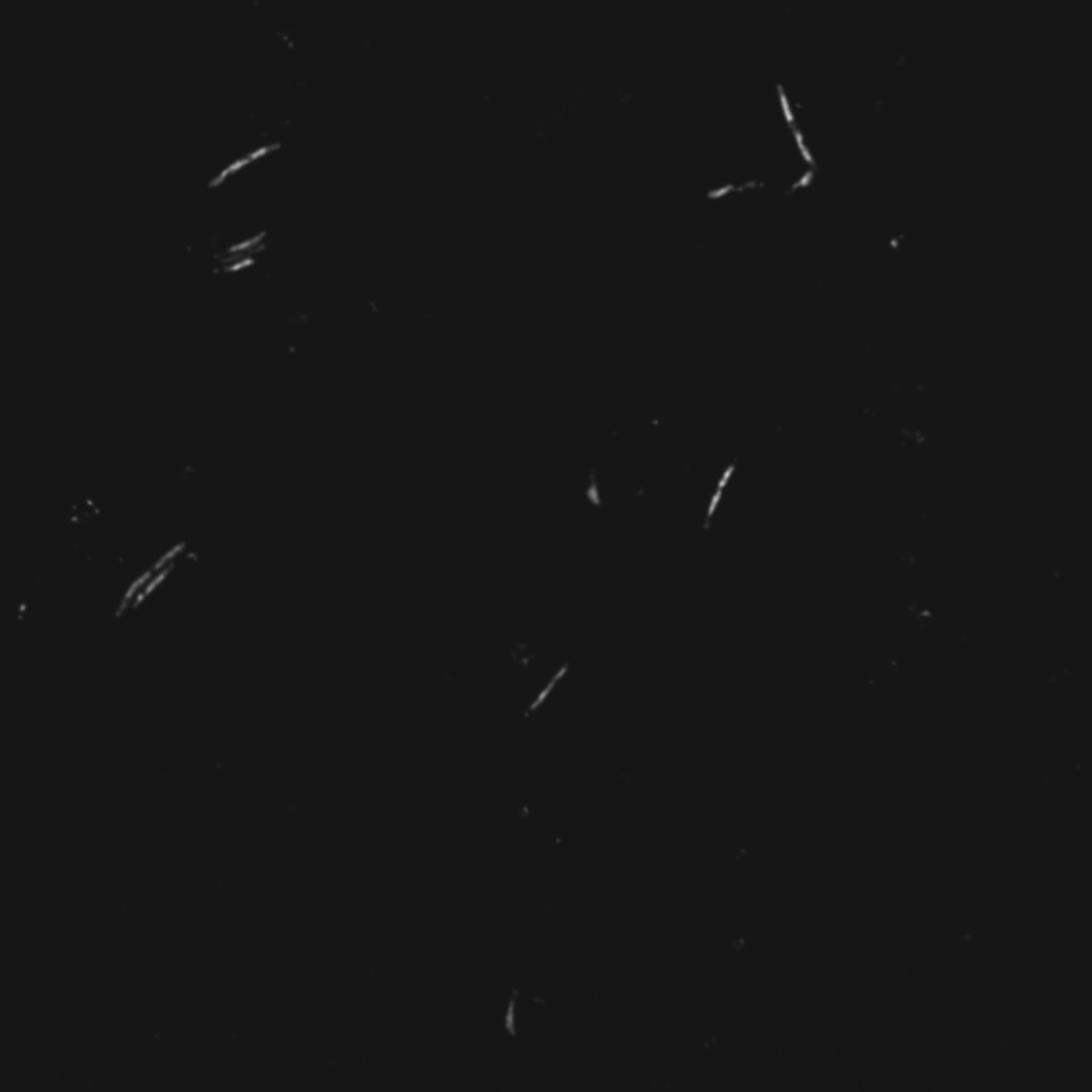

Supplement: S1 File — (ZIP) [file pone.0263975.s001.zip › raw data/microscopy/localization/RmlA_PI_z_2_decon - Z=10 C=1.jpg]

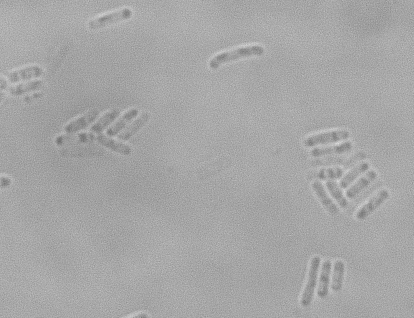

Supplement: S1 File — (ZIP) [file pone.0263975.s001.zip › raw data/microscopy/morphology/dpkng_tc_0.2.png]

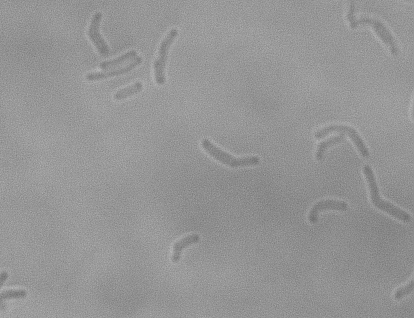

Supplement: S1 File — (ZIP) [file pone.0263975.s001.zip › raw data/microscopy/morphology/lx_2.png]

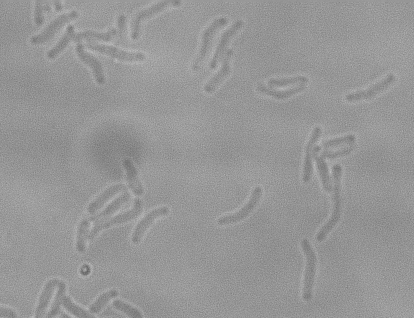

Supplement: S1 File — (ZIP) [file pone.0263975.s001.zip › raw data/microscopy/morphology/lx_tc0.1_1.png]

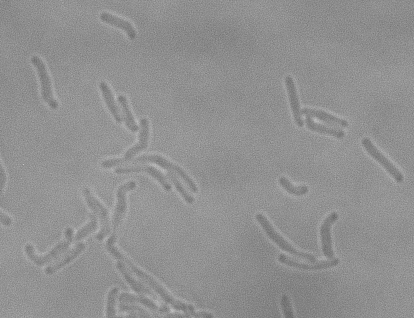

Supplement: S1 File — (ZIP) [file pone.0263975.s001.zip › raw data/microscopy/morphology/lx_tc0.2_3.png]

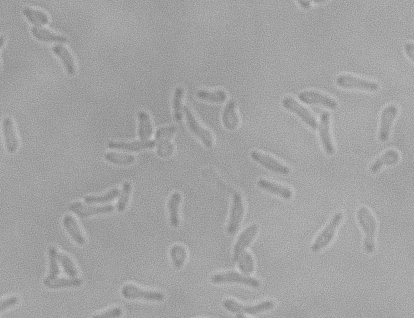

Supplement: S1 File — (ZIP) [file pone.0263975.s001.zip › raw data/microscopy/morphology/pkng rmla tc0.1.png]

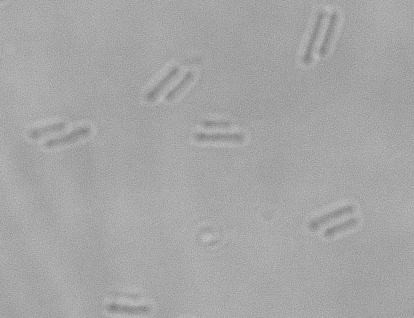

Supplement: S1 File — (ZIP) [file pone.0263975.s001.zip › raw data/microscopy/morphology/pkng tc_0.1.png]

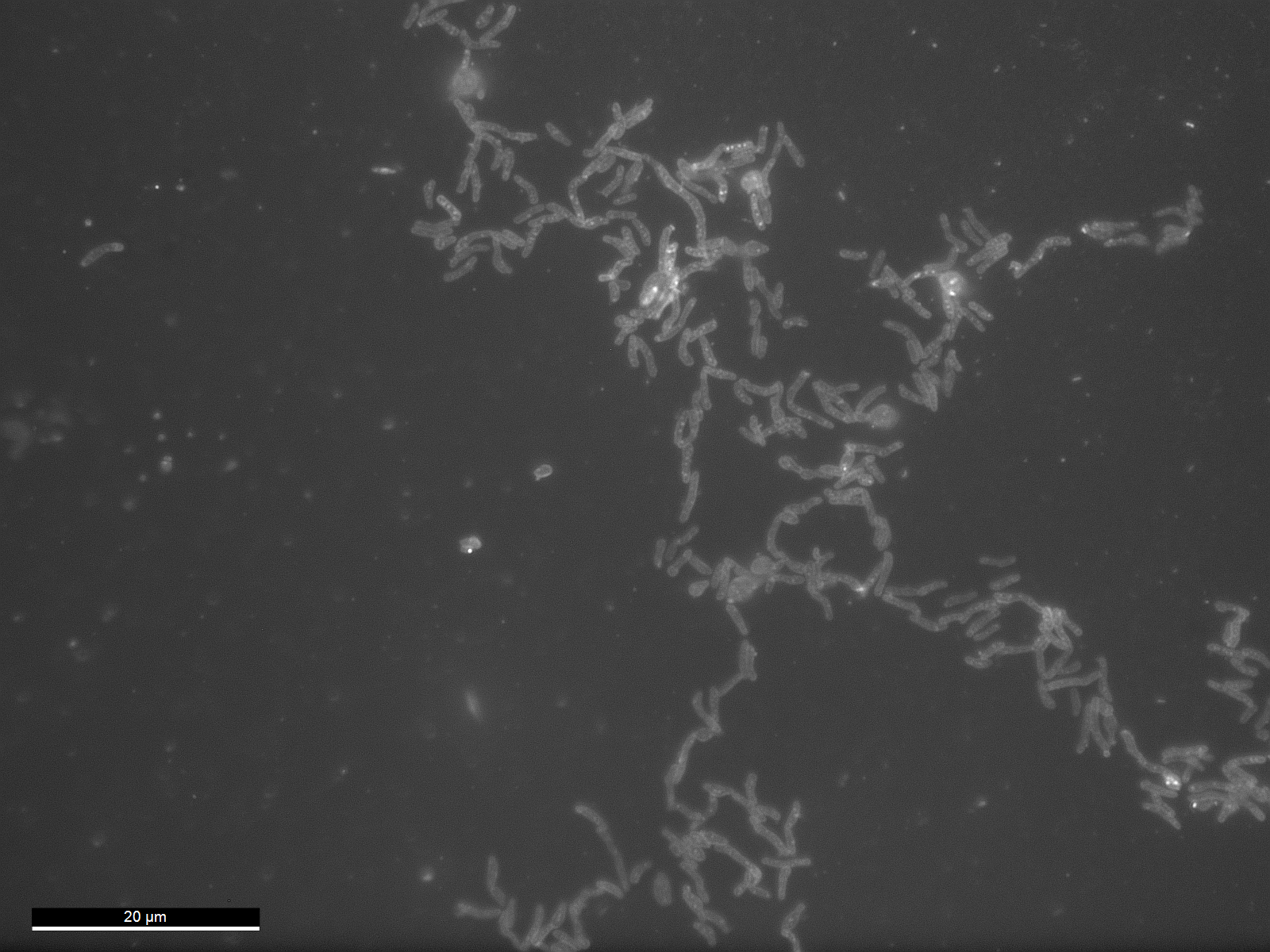

Supplement: S1 File — (ZIP) [file pone.0263975.s001.zip › raw data/microscopy/morphology/pkngrmla_bodipy.tif]

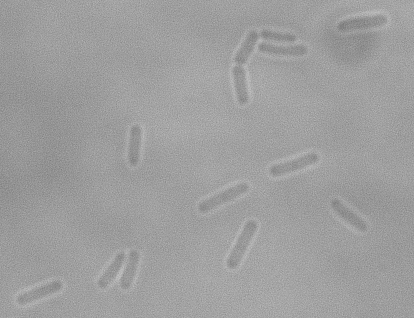

Supplement: S1 File — (ZIP) [file pone.0263975.s001.zip › raw data/microscopy/morphology/pkng_1.png]

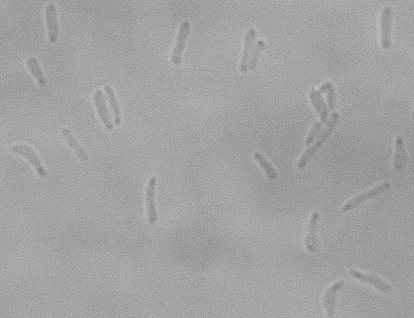

Supplement: S1 File — (ZIP) [file pone.0263975.s001.zip › raw data/microscopy/morphology/pkng_rmla_noind_.png]

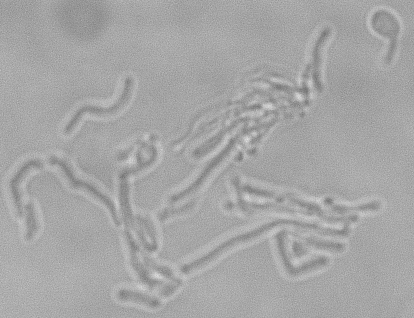

Supplement: S1 File — (ZIP) [file pone.0263975.s001.zip › raw data/microscopy/morphology/pkng_rmla_tc0.2.png]

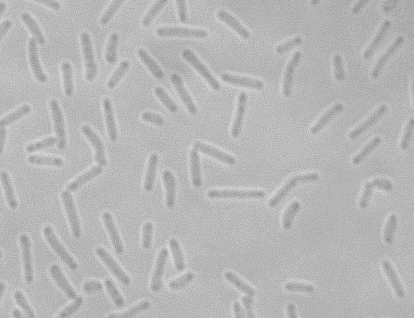

Supplement: S1 File — (ZIP) [file pone.0263975.s001.zip › raw data/microscopy/morphology/wt.png]

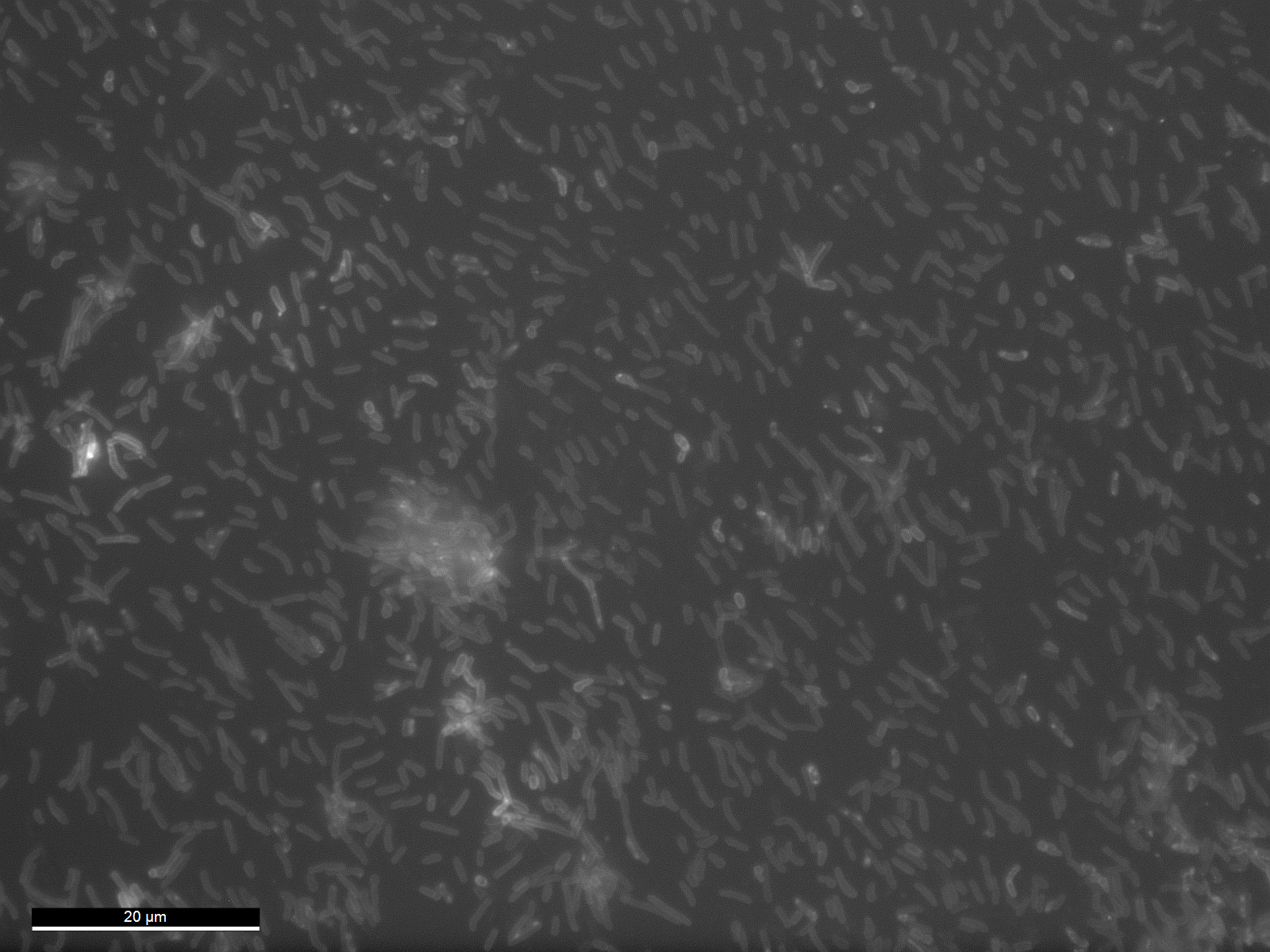

Supplement: S1 File — (ZIP) [file pone.0263975.s001.zip › raw data/microscopy/morphology/wt_bodipy.tif]

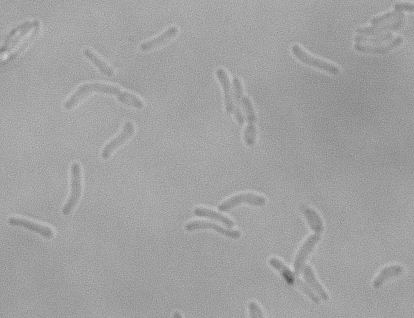

Supplement: S1 File — (ZIP) [file pone.0263975.s001.zip › raw data/microscopy/morphology/wt_rmla tc_0.1.png]

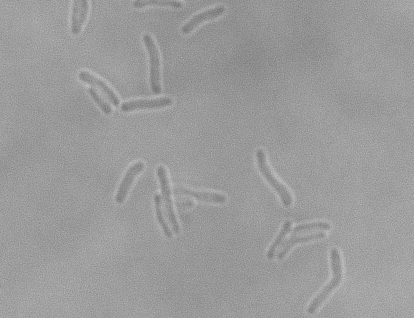

Supplement: S1 File — (ZIP) [file pone.0263975.s001.zip › raw data/microscopy/morphology/wt_rmla.png]

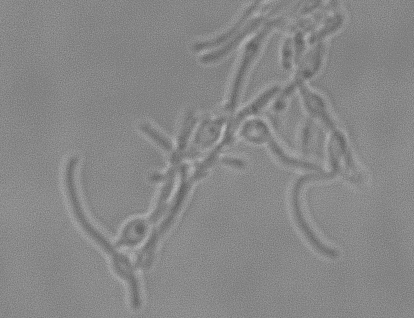

Supplement: S1 File — (ZIP) [file pone.0263975.s001.zip › raw data/microscopy/morphology/wt_rmlA_tc0.2.png]

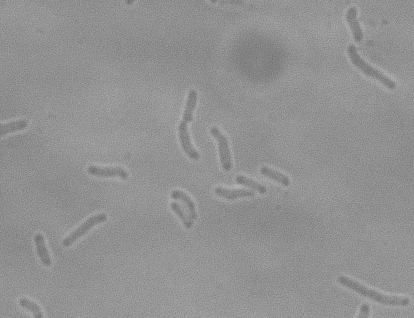

Supplement: S1 File — (ZIP) [file pone.0263975.s001.zip › raw data/microscopy/morphology/wt_tc0.1_2.png]

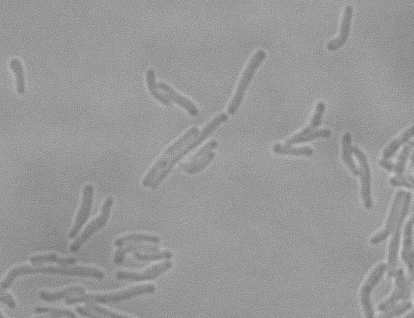

Supplement: S1 File — (ZIP) [file pone.0263975.s001.zip › raw data/microscopy/morphology/wt_tc0.2_6.png]
