# Supplementary material for: The effects of mycobacterial RmlA perturbation on cellular dNTP pool, cell morphology, and replication stress in Mycobacterium smegmatis
Source: PLoS One. 2022 Feb 24;17(2):e0263975. doi: 10.1371/journal.pone.0263975 (PMC8870461; doi:10.1371/journal.pone.0263975)
Supplement: S2 File — (DOCX) [file pone.0263975.s002.docx]

# Request for Change to Authorship

Check to confirm you have read [*PLOS ONE*’s authorship policy](http://journals.plos.org/plosone/s/authorship).

The authorship criteria for *PLOS ONE*, summarized below, are based on those outlined by the International Committee of Medical Journal Editors (ICMJE):

1. Conception and design of the work, acquisition of data, or analysis and interpretation of data
2. Drafting the article or revising it critically for important intellectual content
3. Final approval of the version to be published
4. Agreement to be accountable for all aspects of the work

Authors should meet all of the criteria; the contributions of all authors will be disclosed in the final publication. Any contributions that fall short of the criteria should be named in the Acknowledgments section of the manuscript. It is your responsibility to ensure that anyone named in the Acknowledgments consents to being named.

Check to confirm that all authors (including those to be added or removed) consent to the changes detailed below.

| **Reason for change in author list**  Please briefly describe the reason for adding/removing an author. | The added author, Dániel Molnár, contributed to the experimental work needed for the revision of the manuscript. |
| --- | --- |

# Final manuscript information

| **Manuscript number**  e.g., PONE-D-17-00000 | PONE-D-21-26930R1 |
| --- | --- |
| **Complete author list, in correct order**  Please note any equal contributors with asterisks (*) or hashes (#) | Rita Hirmondó, Ármin Horváth, Dániel Molnár, György Török, Liem Nguyen, Judit Tóth |
| [**Financial Disclosure**](http://journals.plos.org/plosone/s/disclosure-of-funding-sources) – including any additions/deletions necessary due to the change in authorship | This work was supported by the National Research, Development, and Innovation Office, Hungary, OTKA FK124527 and K138318 to JT and PD128254 to RH. Work in L.N. laboratory was supported by National Institutes of Health grants R01AI087903, R21AI119287 and R21AI159770. The funders had no role in study design, data collection and analysis, decision to publish, or preparation of the manuscript. |
| [**Competing Interests**](http://journals.plos.org/plosone/s/competing-interests) – including any additions/deletions necessary due to the change in authorship | The authors have declared that no competing interests exist. |
| [**Acknowledgments statement**](http://journals.plos.org/plosone/s/submission-guidelines#loc-acknowledgments)  Please acknowledge any removed authors if they contributed to the study in any way, as well as members of any author groups who do not meet our authorship criteria. | We thank Krisztina Németh for fluorescence imaging trials. |

# Adding authors

## Individual author addition #1

| **Full name** | Dániel Molnár |
| --- | --- |
| **Email address** | molnar.daniel@ttk.hu |
| **Full affiliation** | 1 Institute of Enzymology, Research Centre for Natural Sciences, Budapest, Hungary  2 Doctoral School of Biology and Institute of Biology, Eötvös Loránd University, Budapest,  Hungary |

| This person contributed to **all** of the following:   1. Conception and design of the work, acquisition of data, or analysis and interpretation of data 2. Drafting the article or revising it critically for important intellectual content 3. Final approval of the version to be published 4. Agreement to be accountable for all aspects of the work |  |
| --- | --- |
| **Specific contributions:** | |
| Conceptualization |  |
| Data Curation |  |
| Formal Analysis |  |
| Funding Acquisition |  |
| Investigation |  |
| Methodology |  |
| Project Administration |  |
| Resources |  |
| Software |  |
| Supervision |  |
| Validation |  |
| Visualization |  |
| Writing – Original Draft Preparation |  |
| Writing – Review & Editing |  |

## Individual author addition #2 (if applicable)

| **Full name** |  |
| --- | --- |
| **Email address** |  |
| **Full affiliation** |  |

| This person contributed to **all** of the following:   1. Conception and design of the work, acquisition of data, or analysis and interpretation of data 2. Drafting the article or revising it critically for important intellectual content 3. Final approval of the version to be published 4. Agreement to be accountable for all aspects of the work |  |
| --- | --- |
| **Specific contributions:** | |
| Conceptualization |  |
| Data Curation |  |
| Formal Analysis |  |
| Funding Acquisition |  |
| Investigation |  |
| Methodology |  |
| Project Administration |  |
| Resources |  |
| Software |  |
| Supervision |  |
| Validation |  |
| Visualization |  |
| Writing – Original Draft Preparation |  |
| Writing – Review & Editing |  |

## Author group addition (if applicable)

| **Group or consortium name** |  |
| --- | --- |
| **Author who represents group** |  |

# Removing authors

## Author removal #1

| **Full name** |  |
| --- | --- |

| This person **did not** contribute to all of the following:   1. Conception and design of the work, acquisition of data, or analysis and interpretation of data 2. Drafting the article or revising it critically for important intellectual content 3. Final approval of the version to be published 4. Agreement to be accountable for all aspects of the work |  |
| --- | --- |
| This person consents to being acknowledged in the published paper. |  |

## Author removal #2 (if applicable)

| **Full name** |  |
| --- | --- |

| This person **did not** contribute to all of the following:   1. Conception and design of the work, acquisition of data, or analysis and interpretation of data 2. Drafting the article or revising it critically for important intellectual content 3. Final approval of the version to be published 4. Agreement to be accountable for all aspects of the work |  |
| --- | --- |
| This person consents to being acknowledged in the published paper. |  |
